# Effect of Pterygium Surgery on Tear Osmolarity

**DOI:** 10.1155/2013/863498

**Published:** 2013-01-20

**Authors:** Kemal Türkyılmaz, Veysi Öner, Mehmet Şahin Sevim, Ali Kurt, Berrak Şekeryapan, Mustafa Durmuş

**Affiliations:** ^1^Department of Ophthalmology, Recep Tayyip Erdoğan University Medical School, 53100 Rize, Turkey; ^2^Department of Ophthalmology, Haydarpasa Numune Research and Education Hospital, 34668 Istanbul, Turkey; ^3^Maya Eye Hospital, 38039 Kayseri, Turkey

## Abstract

*Purpose*. To investigate changes of dry eye test results in patients who underwent pterygium surgery. *Methods*. Seventy-four patients who underwent primary pterygium surgery were enrolled in this study. At the baseline, 3-, 12-, and 18-month visits, measurements of tear osmolarity, BUT, and Schirmer test were performed. The patients were divided into 2 groups: Group 1, which consisted of patients in whom pterygium did not recur, and Group 2, which consisted of patients in whom pterygium recurred after surgery. *Results*. The patients in Group 1 had lower tear osmolarity levels after surgery than those at baseline (all *P* < 0.001). In Group 2 the tear osmolarity levels did not differ from baseline after 18 months (*P* = 0.057). The prevalence rates of dry eye syndrome (DES) were lower than that at baseline and 18 months after surgery in Group 1 (*P* = 0.002). In Group 2, the incidence of DES was lower after 3 months than at baseline (*P* = 0.03) but was similar to the baseline rate after 12 and 18 months (both *P* > 0.05). *Conclusions*. Anormal tear film function associated with pterygium. Pterygium excision improved tear osmolarity and tear film function. However, tear osmolarity deteriorated again with the recurrence of pterygium.

## 1. Introduction

Pterygium is a common disease of the ocular surface characterized by the invasion of fibrovascular tissue from the bulbar conjunctiva onto the cornea. It can cause chronic ocular irritation, induced astigmatism, tear film disturbances, and decreased vision secondary to growth over the visual axis [1]. Although the exact etiology of pterygium is unknown, exposure to ultraviolet (UV) radiation is thought to be the major environmental risk factor [2]. Age, hereditary factors, sunlight, chronic inflammation, microtrauma, and dry eye are other possible contributing factors [3–6]. The most commonly accepted treatment for pterygium is surgical excision. However, the rate of recurrence after surgery is high [7]. Several studies have used tear function tests, such as the Schirmer test or tear breakup time (BUT), to investigate the relationship between pterygium and dry eye syndrome (DES), with conflicting results [5, 8, 9]. In addition, a very few studies have evaluated the effects of the excision of pterygium on tear function [10, 11].

Various methods (i.e., the BUT, Schirmer, and mucus fern tests) are available for the investigation of DES. However, these tests are not always reliable, and none of them alone is sufficient for diagnosis [12]. Elevated tear osmolarity has recently been shown to be a reliable indicator of DES, and it has been proposed as a potential gold standard for diagnosis [12, 13]. However, to the best of our knowledge, measurement of tear osmolarity has not been used to investigate the relationship between surgical excision of pterygium and DES. Therefore, in this study we aimed to investigate the changes in tear osmolarity, breakup time (BUT), and Schirmer test results in patients who had undergone pterygium surgery and to evaluate how these parameters changed when pterygium recurred after primary surgery.

## 2. Materials and Methods

Seventy-four eyes of 74 patients that underwent primary pterygium surgery were enrolled consecutively in this prospective study. Clinical visits were made at baseline (before surgery), 1, 7, and 15 days, and 3, 12, and 18 months after surgery. At the baseline, 3-, 12-, and 18-month visits, measurements of tear osmolarity and BUT and the Schirmer test were performed by the same investigator (KT) for each patient. The presence of fibrovascular tissue with a horizontal length from limbus to cornea of ≥2 mm (measured by slit lamp biomicroscopy) was accepted as pterygium and treated by pterygium surgery. Extent of its invasion onto the cornea was assessed for determining severity of pterygium. Fibrovascular growth onto the cornea of >0.5 mm recorded during the postoperative follow-up period was accepted as recurrence of pterygium. The patients were divided into 2 groups: Group 1, which consisted of patients in whom pterygium did not recur, and Group 2, which consisted of patients in whom pterygium recurred after surgery. All patients were informed about the study procedure and gave written informed consent to participate. This study followed the Tenets of the Declaration of Helsinki and was approved by the Recep Tayyip Erdogan University Medical Faculty Ethics Committee.

Each patient underwent a standard ophthalmological examination to exclude patients with ocular or extraocular diseases other than pterygium that could affect tear film function, such as blepharitis, ocular allergy, thyroid diseases, lacrimal system disorders, diabetes, collagen diseases, and use of any topical or systemic drug during the 3-month period before the examination.

### 2.1. Surgical Procedures

After topical and subconjunctival administration of 2% lidocaine for anesthesia, the head of the pterygium was separated and dissected away from the cornea. The pterygium was resected, the episcleral and Tenon's tissues were dissected away from the overlying sclera, and the dissociated edges of the conjunctiva were closed with 10/0 polyglycolic acid suture, leaving a 4 mm area of bare sclera. At the end of the surgery, 0.3% tobramycin ointment was applied topically before patching. Prednisolone acetate (1%) and 0.3% tobramycin were applied topically 4 times daily for 2 weeks. The sutures were removed 7 days after surgery.

### 2.2. Tear Film Function Tests

The Schirmer test was performed without topical anesthesia. The length of the strip that was wet after 5 minutes was measured and accepted as the test result. The BUT was measured using fluorescein and a slit lamp with cobalt blue illumination. The average value of 2 consecutive measurements was used for analysis. The BUT was evaluated at least 30 minutes after the Schirmer test.

### 2.3. Tear Osmolarity

Tear osmolarity was measured using the TearLab Osmolarity System (TearLab Corp., San Diego, CA, USA) at least 30 minutes after the tear function tests for each patient. When the system was ready, the patient was requested to look up, and a handled pen with a chip test card that could serve as a laboratory assay mounted on its tip was touched to the inferior tear meniscus located above the lower eyelid. After the green light on the pen went out, indicating the conclusion of the tear-collection process, the pen was placed on the TearLab Reader. The code on the chip test card was entered into the TearLab Reader, and the results of the measurement process were obtained within a maximum of 30 seconds. Values of >312 mOsm/L were considered indicative of DES [14].

### 2.4. Statistical Analysis

Statistical analyses were performed using SPSS version 16.00. All variables were distributed normally and expressed as the mean ± standard deviation. Categorical variables were compared between the groups using the chi-square test. The Friedman test and paired *t*-test were used for comparisons within each group. The McNemar test was used to compare prevalence rates within each group. The level of statistical significance was set at *P* < 0.05.

## 3. Results

There were 50 patients (32 male and 28 female) in Group 1 and 24 patients (15 male and 9 female) in Group 2. All recurrences of pterygium occurred between the 3rd and 18th postoperative months. The mean age was 50.5 ± 6.8 (range, 31 to 59) years in Group 1 and 50.2 ± 6.9 (range, 33 to 58) years in Group 2. The patients' age and sex ratio did not differ significantly between the groups.

The comparisons of tear osmolarity, BUT, and Schirmer test results between the groups during the follow-up period are shown in Table 1. Tear osmolarity levels changed significantly over the follow-up period within study Group 1 (all *P* < 0.001). The patients in Group 1 had significantly lower tear osmolarity levels 3, 12, and 18 months after surgery than at baseline (all *P* < 0.001). In contrast, in Group 2 the tear osmolarity levels decreased significantly between baseline and 3 months after surgery (*P* = 0.032) but returned to baseline levels at the 12-month follow-up visit (*P* = 0.707) and did not differ significantly from baseline after 18 months (*P* = 0.057).

Fourteen of 50 eyes (28.0%) in Group 1 and 8 of 24 eyes (33.3%) in Group 2 exhibited DES preoperatively (*P* > 0.05). The prevalence rates of DES were significantly lower than at baseline 3 (6.0%), 12 (6.0%), and 18 (8.0%) months after surgery in Group 1 (*P*1 and *P*2 = 0.001 and *P*3 = 0.002). In Group 2, the incidence of DES was significantly lower after 3 months (8.3%) than at baseline (*P* = 0.03) but was similar to the baseline rate after 12 (29.1%) and 18 months (29.1%) (both *P* > 0.05).

The BUT results changed significantly over the follow-up period within Group 1 (*P* < 0.001). The patients had significantly higher BUT values 3, 12, and 18 months after surgery than at baseline (all *P* < 0.001). However, the results of the BUT test did not change significantly within Group 2 (*P* > 0.05). In addition, the results of the Schirmer test did not change significantly within either group (both *P* > 0.05).

Preoperatively, the length of the fibrovascular tissue correlated with the tear osmolarity and BUT (*r* = −0.268, *P* = 0.021 and *r* = −0.248, *P* = 0.033, resp.). However, the length of the fibrovascular tissue did not correlate with the result of the Schirmer test (*P* > 0.05).

There was no correlation between the length of the recurrent fibrovascular tissue and the results of the dry eye tests 18 months after surgery in the recurrent pterygium group (all *P* > 0.05).

## 4. Discussion

This study has demonstrated that tear osmolarity and BUT values improved significantly after primary pterygium excision in Group 1. On the other hand, although tear osmolarity levels were significantly better 3 months after surgery in Group 2, they deteriorated and exceeded baseline levels after 12 and 18 months. In addition, the incidence of DES significantly decreased after excision of pterygium in both groups and increased again only in cases of recurrent pterygium. Furthermore, the BUT values of Group 2 and Schirmer test results of both groups were similar to baseline levels throughout the follow-up period.

Tear hyperosmolarity has been identified as an important factor in the pathogenesis of DES and has recently been included as a part of the definition of dry eye [15]. The preocular tear film layer is the eye's first line of defense against environmental insults such as dryness and UV exposure. Therefore, some authors have thought that impairment of tear function could be a risk factor for diseases caused by UV exposure, including pterygium [8, 9]. Conversely, the reverse mechanism, that is, that conjunctival, corneal, or eyelid changes associated with pterygium disturb tear film function, has also been proposed [16].

In the present study, we found statistically significant differences in the mean tear osmolarity values within the groups over time. However, these changes (ranging from 300 to 306 mOsm/L) may not be clinically relevant. We speculated that changes in the prevalence of dry eye might be more important than the differences in the mean tear osmolarity values. According to the cut-off value for tear osmolarity, 28% of the patients in Group 1 had DES before surgery. The prevalence of DES decreased after surgery, and only 8% of the patients had DES 18 months after pterygium removal. In contrast, 33.3% of the patients in Group 2 had DES before surgery based on their tear osmolarity values. The prevalence of DES decreased after surgery, and only 8.3% of the patients had DES 3 months after surgery. However, 18 months after surgery the prevalence of DES in Group 2 (29.1%) had rebounded almost to the preoperative level.

In summary, the prevalence of DES according to the tear osmolarity level decreased significantly after surgical excision of pterygium but increased again after recurrence of pterygium. Accordingly, we concluded that the presence of pterygium seems to cause DES.

Several studies have investigated the relationship between pterygium and changes in tear film function [5, 8, 9, 17, 18]. Pterygium has been shown to be associated with abnormal tear film function, such as a shortened tear breakup time (BUT) or abnormal mucus fern patterns [8, 9, 17]. However, conflicting results have also been reported [5, 18]. In 2 previous studies with follow-up periods of 1 [10] and 2 [11] months, the results of the BUT and mucus fern tests, but not the Schirmer test, improved significantly over their respective baseline values following pterygium excision. Conversely, another study with a 6-month follow-up period found no difference between the Schirmer and BUT test results at baseline and those obtained 1 and 6 months after surgery [19].

We believe that these contradictory results may have been obtained because the methods that were used to evaluate tear function were not objective and quantitative. The present study has shown that although the BUT test results improved after surgical treatment of pterygium with no recurrence, the Schirmer test results did not change. Therefore, we can speculate that the quantity of the tear film in patients with pterygium is adequate but that its quality or composition is abnormal. In addition, to the best of our knowledge, this is the first time that tear osmolarity levels have been used to determine the composition of the tear film in patients with pterygium, and we revealed that it improved after surgical treatment and remained stable for 18 months after surgery so long as the pterygium did not recur.

UV-mediated genetic trauma may affect the expression of cytokines, such as interleukin (IL)-6 and IL-8, in patients with pterygium [20]. IL-6 and IL-8 can induce the production of matrix metalloproteinases (MMPs), which are localized to the advancing edges of pterygium [21, 22]. The release of IL-6, IL-8, and MMPs into the tear film may lead to ocular surface damage and tear film instability, ultimately resulting in epithelial cell apoptosis, goblet cell loss, a reduction in mucus secretion, and tear hyperosmolarity [15]. Eventually, a vicious cycle develops in which tear hyperosmolarity itself stimulates MMP expression and thus leads to ocular surface inflammation [23].

Tear osmolarity can be measured by various methods that rely on changes in the freezing point or electrical conductivity of the tears [24]. In some methods, prolonged ocular contact during the collection of the tear sample elevates tear secretion and decreases tear osmolarity [25]. In this study, we used the TearLab Osmolarity System, which uses a gold electrode inside the channel to measure the electrical impedance [24]. This is a fast and simple technique that minimizes reflex tear production and evaporation of the tear sample [26]. The system is noninvasive, and the measurement takes less than a minute. Only 50 *μ*L of tear sample is collected, by passive capillary movement, and the effect of vaporization is thus minimized.

A meta-analysis found that the recurrence rate after pterygium surgery was higher when the bare sclera technique was used than when a limbal conjunctival autograft was employed [27].

The recurrence rate of pterygium ranges from 24% to 89% when treated with the bare sclera technique [28–30] but from 1.6% to 33% when treated with conjunctival autografting [31]. Amniotic membrane grafting has been used as an alternative to limbal conjunctival autografting, as the recurrence rate does not differ significantly between these techniques [27]. The recurrence rate in the present study, which employed the bare sclera technique, was 32.4%. We believe that the use of limbal autografting would have decreased the recurrence rate.

One limitation of our study is that there were gaps between visits, the longest of which was 9 months. Therefore, we do not know for certain the earliest time at which tear osmolarity increased or pterygium recurred.

We reasonably supposed that pterygium recurrence may lead to dry eye because the pterygium disturbs tear function. Conversely, it can be speculated that dry eye may cause the recurrence of pterygium or that more severe underlying dry eye may contribute to recurrence. However, the essentially equal results for the tear osmolarity, BUT, and Schirmer test in our 2 groups prior to surgery provide strong evidence that it is pterygium recurrence that leads to dry eye.

In conclusion, this study revealed that tear hyperosmolarity and abnormal tear film function are associated with pterygium. Pterygium excision improved tear osmolarity and tear film function. However, tear osmolarity deteriorated again with the recurrence of pterygium. Therefore, we infer that pterygium seems to cause DES and that surgical removal of pterygium alleviates pterygium-related DES.

## Figures and Tables

**Table 1:** The comparisons of the tear osmolarity, break-up time (BUT), and Schirmer test results within the groups during the follow-up period (mean ± SD).

	Group 1 (*n* = 50)	Group 2 (*n* = 24)
Tear osmolarity (mOsm/L)		
Baseline	304.9 ± 8.0	304.0 ± 11.8
3rd month	301.8 ± 8.2	301.6 ± 11.7
12th month	300.0 ± 8.8	305.0 ± 11.2
18th month	300.1 ± 8.6	306.7 ± 11.7
BUT (second)		
Baseline	10.5 ± 3.0	10.3 ± 3.7
3rd month	11.8 ± 3.8	10.7 ± 3.9
12th month	11.9 ± 3.4	11.2 ± 3.7
18th month	11.8 ± 3.5	11.1 ± 3.9
Schirmer test (mm)		
Baseline	12.5 ± 3.6	12.0 ± 3.9
3rd month	12.5 ± 3.5	12.2 ± 3.1
12th month	12.7 ± 3.9	12.5 ± 3.6
18th month	12.9 ± 3.6	12.7 ± 3.6

Group 1 includes patients with no recurrence of pterygium after primary pterygium surgery.

Group 2 includes patients with recurrence of pterygium after primary pterygium surgery.
